# Effect of Bioactive Compounds from Pumpkin Powder on the Quality and Textural Properties of Shortbread Cookies

**DOI:** 10.3390/foods12213907

**Published:** 2023-10-25

**Authors:** Aliona Ghendov-Mosanu, Natalia Netreba, Greta Balan, Daniela Cojocari, Olga Boestean, Viorica Bulgaru, Angela Gurev, Liliana Popescu, Olga Deseatnicova, Vladislav Resitca, Carmen Socaciu, Adela Pintea, Tamar Sanikidze, Rodica Sturza

**Affiliations:** 1 Faculty of Food Technology, Technical University of Moldova, 9/9 Studentilor St., MD-2045 Chisinau, Moldova; aliona.mosanu@tpa.utm.md (A.G.-M.); olga.boestean@tpa.utm.md (O.B.); viorica.bulgaru@tpa.utm.md (V.B.); angela.gurev@chim.utm.md (A.G.); liliana.popescu@tpa.utm.md (L.P.); olga.deseatnicova@toap.utm.md (O.D.); vladislav.resitca@adm.utm.md (V.R.); rodica.sturza@chim.utm.md (R.S.); 2 Department of Preventive Medicine, “Nicolae Testemitanu” State University of Medicine and Pharmacy, 165 Stefan cel Mare Blvd., MD-2004 Chisinau, Moldova; greta.balan@usmf.md (G.B.); daniela.cojocari@usmf.md (D.C.); 3 Faculty of Veterinary Medicine, University of Agricultural Sciences and Veterinary Medicine, 3–5 Calea Manasturs St., 4003724 Cluj-Napoca, Romania; csocaciudac@gmail.com (C.S.); apintea@usamvcluj.ro (A.P.); 4 Faculty of Exact and Natural Sciences, Javakhishvili Tbilisi State University, 1 Ilia Chavchavadze Ave., Tbilisi 00186, Georgia; tsanikidze@tsmu.edu

**Keywords:** *Cucurbita moschata*, shortbread cookies, biologically active compounds, antioxidant activity, antimicrobial activity, texture, sensory quality, physicochemical quality

## Abstract

The problem of food with functional ingredients, characterized by low energy intake and a variety of phytonutrients with biological activity, is one of the concerns of the population. The objectives of this study were to investigate the effect of pumpkin powder and its bioactive components on the quality, color and textural properties of shortbread cookies. In the drying process of pumpkin powder (*Cucurbita moschata*) at 60 ± 2 °C, the physicochemical parameters did not change significantly in relation to fresh pulp. The chromatic parameters L*, a* and b* showed that the pumpkin powder was brighter than the pulp, with a greater presence of yellow pigments. Pumpkin powder presented a rich source of bioactive compounds (polyphenols flavonoids, carotenoids) with an antioxidant potential of 161.52 mmol TE/100 g DW and 558.71 mg GAE/100 g DW. Antimicrobial activity against Gram-positive (*Staphylococcus aureus*, *Bacillus cereus*), Gram-negative (*Escherichia coli*, *Salmonella Abony* and *Pseudomonas aeruginosa*) bacteria and high antifungal activity against *Candida albicans* were attested. The sensory, physicochemical, texture parameters and color indicators of shortbread cookies with yellow pumpkin powder (YPP) added in a proportion of 5–20% were analyzed. The optimal score was given to the sample of 15% YPP. The use of 15–20% YPP contributed to improved consistency due to the formation of complexes between starch and protein.

## 1. Introduction

Pumpkin (*Cucurbita moschata*), widely cultivated in different climatic zones, famous for its nutritional value and health-promoting effects, is consumed in abundance as a functional food and as a medicine for the treatment of various health conditions [1,2,3]. Pumpkin seeds, peel, pulp, flowers and leaves contain compounds characterized by high bioactivity, such as polysaccharides, proteins, polyphenols, carotenoids, phytosterols, vitamins and minerals, which positively affect human health [4,5,6].

The qualitative and quantitative profile of biologically active compounds in pumpkin seeds, peels and cores depends on several factors: genotype [7], cultivation method, maturity degree, storage conditions and duration, processing method, drying and functional compound extraction process [8,9]. Several studies have shown that the antioxidant components contained in pumpkin inhibit free radicals and reduce the risk of cancer, cardiovascular and neurodegenerative diseases [10,11].

Pumpkin pulp is a source of dietary fiber, especially pectin, with low energy value, useful in lowering blood glucose levels [2]. The water content in pumpkin pulp varies from 88 to 96%, and the caloric content per 100 g of fresh material varies, on average, from 15 kcal to 46 kcal [12]. Low molecular weight polysaccharides (3.5 kDa), extracted from pumpkin pulp, inhibit free radicals and reduce oxidative stress [13]. Pumpkin pulp and seed extracts showed antibacterial, fungicidal and antioxidant potential [14,15]. Research has shown that pumpkin pulp flour had hypoglycemic and hypolipidemic effects [16]. The antidiabetic activity of pumpkin powder was demonstrated by reducing blood glucose with increased plasma insulin in alloxan-induced diabetic mice [17].

Despite its valuable phytonutrient content and health benefits, pumpkin is used little in the Republic of Moldova’s food industry. One of the causes is the difficulty in processing pumpkin and creating storage spaces with appropriate conditions. These impediments can be reduced by using fruits and vegetables, including pumpkin, in dehydrated or powdered form. The powders, obtained by the correct dehydration of fresh fruits and vegetables, as well as byproducts resulting from their processing, are a concentrated form of biologically active compounds that are bioaccessible, which can be applied in the production of foods with increased biological potential [18,19]. Furthermore, it was determined that drying the pumpkin at higher temperatures did not reduce the antioxidant activity of the pulp [20].

The *β*-carotene content of 3934.02 μg/100 g DW determined in pumpkin powder was higher than in peels and seeds [14]. Pumpkin powder extracts showed increased antioxidant effect due to the high content of biologically active compounds [4]. The total content of carotenoids determined by the researchers in the pumpkin pulp powder was 35.2 mg/100 g DW, and of *β*-carotene, 6.18 mg/100 g DW. According to research [21], pumpkin pulp extracts showed antioxidant, antiproliferative activities and stronger antibacterial potential than pumpkin peel extracts, against three bacterial species (*Escherichia coli*, *Staphylococcus aureus* and *Pasteurella multocida*). The ones presented confirm that pumpkin powder, due to its phytochemical content and functional properties, could be used as a potential source of nutraceuticals in food production [22,23].

Nutraceutical is an umbrella term that includes foods, food parts or dietary supplements that provide physical or protective benefits against chronic diseases. [24]. The bioactive components of pumpkin pulp, such as polyphenols, fibers, polysaccharides, proteins, lipids, amino acids, carotenoids (precursors of vitamin A), vitamins (vitamin C, vitamin B2, vitamin E), minerals (potassium, calcium, magnesium, selenium) [4,25], have been shown to be effective nutraceuticals in the treatment of oxidative stress-related healing disorders, including allergies, Alzheimer’s disease, cardiovascular disease, cancer, diabetes, ocular, immune, inflammatory and Parkinson’s diseases, as well as obesity [26].

Pumpkin powder is an exceptional raw material and natural colorant for nectars, juices, fermented drinks, jams, marmalades, purees, teas, etc. Fortifying foods with pumpkin powder gives them sweet taste, texture, color, volume and low-calorie content [12]. Recent research has shown that pumpkin powder, rich in minerals, vitamins, proteins and antioxidants, can replace white flour in bakery products [27,28], which, in addition to providing substantial nutrients, changes the texture and sensory properties of the fortified product [29].

An excellent food matrix for the functional ingredients of pumpkin powder is cookies [25], which are preferred by all categories of consumers, especially children.

Therefore, in addition to fresh consumption, it is important to implement technologies to develop new nutraceuticals from the beneficial health constituents of pumpkin, as well as to develop fortified food products that are attractive to consumers.

The objective of the present study was to investigate the effect of pumpkin powder and its bioactive components on the quality, color and textural properties of shortbread cookies.

## 2. Materials and Methods

### 2.1. Chemical Materials

Folin–Ciocalteu reagent was purchased from Merck (Darmstadt, Germany). Gallic acid (98%), quercetin (>95%), methanol (≥99.9%), tert-butyl methyl ether (≥99.9%), hydrochloric acid (37%), ethyl acetate (≥99.5%), petroleum ether, sodium hydroxide (97%), phenolphthalein, sodium nitrite (≥97.0%), aluminum chloride, 2,2-diphenyl-1-picrylhydrazyl, (±)-6-hydroxy-2,5,7,8-tetramethylchromane-2-carboxylic acid, sodium citrate (≥99.0%), trisodium citrate, silver nitrate (≥99.0%), hexane reagent (>99%), boric acid (≥99.5%), hydrochloric acid (37%), KjTabs VST, sulfuric acid (95.0–98.0%), potassium hydroxide (≥85%), n-octanol (≥99%), anhydrous acetone (≥99.5%) were obtained from Sigma (Darmstadt, Germany; Tokyo, Japan; Shanghai, China). Lutein and *β*-carotene were purchased from Extrasynthese (Lyon, France). A Specord 200 Plus spectrophotometer (Jena, Germany) was used for spectrophotometric measurements.

### 2.2. Biological Material

Fully ripe pumpkins of the variety butternut squash (species *Cucurbita moschata*), harvested in September, were procured from the company “S.C. S&S COM” S.R.L. (Costesti, Moldova), located in the northern part of the Republic of Moldova (46.8677787780761700 latitude, 28.7686119079589840 longitude and altitude of 80 m above sea level). The fruits were washed and cut into two portions; the fruit matrix and seeds were scooped out.

### 2.3. Characterization of Pumpkin Fresh Pulp and Powder

Pumpkin halves were cut into slices. The slices were peeled and grated into shreds. The shreds of pumpkin were dried in a laboratory drying oven, model SLW 115 SMART, with forced air circulation at a constant temperature of 60 ± 2 °C and relative humidity of 60–65%. The drying process was carried out for 24 h, until they reached a safe moisture content level, under 8% (wet basis). The average water content was periodically measured with a halogen moisture analyzer (model HG53, from Mettler Toledo, Columbus, OH, USA), which was previously calibrated in terms of optimal operating parameters for vegetable foods. The dry shreds of pumpkin were crushed to powder with a particle size of 175 ± 15 μm and then sieved. The pumpkin powder was packed in a hermetic box for subsequent use in this study and the preparation of cookies.

#### 2.3.1. Color Analysis of Fresh Pumpkin Fruits, Pumpkin Powder and Shortbread Cookies Using the CIELAB Method

The CIELab parameters, L*, a* and b*, were measured using a Chroma Meter CR-400/410 colorimeter (Konica Minolta, Tokyo, Japan) according to the method in [30]. Each sample was analyzed at three distinct points, measuring variations in brightness (L*), red/green component (a*) and yellow/blue component (b*).

Equation (1) was used to determinate the overall difference in color—${\Delta E}^{*}$:

(1) $${\Delta E}^{*}=\surd (L_{i}^{*}-L_{0}^{*}{)}^{2}+(a_{i}^{*}-a_{0}^{*}{)}^{2}+(b_{i}^{*}-b_{0}^{*}{)}^{2}$$

where

$L_{0}^{*},a_{0}^{*}\;and\;b_{0}^{*}—$the values of the control sample;

$L_{i}^{*},a_{i}^{*}\;and\;b_{i}^{*}—$the values of the samples with pumpkin powder.

#### 2.3.2. Physicochemical Analysis of Fresh Pumpkin Fruits and Pumpkin Powder

The moisture content (MC) was determined by measuring the weight loss due to evaporation of water (AOAC, 2012). The total soluble solids (TSS) of the pumpkin pulp were measured using a handheld Brix refractometer at 0–32° (AOAC, 2012). The pH was measured using a pH meter (TESTO 206-ph2, Pruszkow, Poland) calibrated with buffer solutions pH 4.0 and 7.0, directly immersing the electrode in the beaker containing the sample macerated with distilled water, according to the AOAC (2012) method [31]. The ash content (AC) was estimated using the method described by Ranganna [32]. The titratable acidity (TA) was estimated by titrating a known volume of sample against standard 0.1 N NaOH using phenolphthalein as an indicator. The results were expressed in % citric acid [32,33]. The fat content (FC), protein content (PC) and total fiber content (TFC) of the samples were determined using the method reported by the AOAC (2012) [31]. The FC was gravimetrically quantified after hexane extraction from the dehydrated sample using the Soxhlet method in a SER148 Solvent Extraction Unit (VELP Scientifica, Monza, Italy) [33]. The PC was analytically estimated by determining the amount of total nitrogen content with a conversion factor of 6.25, using the Kjeldahl method in a UDK129 (VELP Scientifica, Italy). The method for determining TFC in FIWE Raw Fiber Extractors (VELP Scientifica, Italy) involves a series of chemical treatments to remove all soluble and easily digestible components from the sample. The remaining fiber components are then washed, dried and weighed as the residual fiber component. The weight of the TFC is calculated as a percentage of the initial weight of the food sample.

### 2.4. Extract Characterization

For sample extraction, 1 g of pumpkin powder was extracted by shaking the sample 1 min at 2000 rpm in a vortex mixer VX-200 (Labnet, Edison, NJ, USA) with 10 mL of acidified methanol (85:15 *v*/*v*, MeOH:HCl). Ultrasonic-assisted extraction (ISOLAB Laborgeräte GmbH, Eschau, Germany) was performed at a frequency of 37 kHz for 30 min; then, the mixture was centrifuged at 3000 rpm for 10 min. After filtration, the total polyphenol content (TPC), total flavonoid content (TFC) and antioxidant activity by reaction with DPPH radical were determined. The extract was stored in glass bottles at 4.0 ± 1.0 °C, in the dark.

#### 2.4.1. Total Polyphenols Using Folin–Ciocalteu

Folin–Ciocalteu reagent was used to determine the total polyphenol content (TPC), according to the method described by Waterman and Mole [34]. The results were calculated from a calibration curve using gallic acid (0–10 mg/L, y = 0.5056x + 0.0650, R^2^ = 0.9977) and expressed in equivalents of gallic acid per 100 g of dried weight (DW) of pumpkin powder (mg GAE/100 g DW).

#### 2.4.2. Total Flavonoids

Total flavonoid content (TFC) in pumpkin extract was determined spectrophotometrically according to Zhishen et al. [35]. The flavonoid–aluminum complex was detected at 510 nm. The quercetin calibration curve was used (0–0.250 mg/L, y = 0.7038x + 0.0004, R^2^ = 0.9955). The results were expressed in quercetin equivalents per 100 g dry weight (DW) of pumpkin powder (mg QE) /100 g DW).

#### 2.4.3. Total Carotenoids

The total carotenoid content (TCC) was determined following the modified method described by Ghendov-Mosanu et al. [36]. The plant material (3 g) was extracted with a mixture of methanol/ethyl acetate/petroleum ether (1: 1: 1, *v/v/v*). After filtering the extract, the residue was reextracted twice using the same solvent mixture. TCC was determined using the spectrophotometric method. The absorption spectrum was plotted and the TCC was measured at the maximum absorbance wavelength (450 nm).

#### 2.4.4. Separation of Carotenoids Using Reverse-Phase High-Performance Liquid Chromatography (RP-HPLC)

Carotenoids were separated using RP-HPLC, on an LC-20AT equipped with an SPD-M20A diode array detector (DAD) (Shimadzu Corporation, Kyoto, Japan), according to the method described by Ghendov-Moșanu et al. [18]. Saponified carotenoid extracts were used for this analysis. Carotenoids were identified based on their retention times and absorption spectra, compared with those of available standards (violaxanthin, lutein, *β*-cryptoxanthin, all-*trans-β*-carotene) and the available literature data. For the quantification of *β*-carotene and lutein, external eight-point calibration curves were constructed in the range 1–100 μg/mL. The regression equations were y = 481,115x − 43,279, correlation coefficient: R^2^ = 0.9985 for *β*-carotene, and, respectively, y = 415,811x − 17,763 and R^2^ = 0.9996 for lutein. The other compounds in the extract were determined using either the curve for lutein (luteoxanthin and violaxanthin), or that of *β*-carotene (*β*-cryptoxanthin and *cis-β*-carotene), Table 1.

#### 2.4.5. Antioxidant Activity, DPPH Assay

The antioxidant activity of the pumpkin extract was determined using the DPPH free radical method, according to Brand-Williams et al. [37]. The calibration curve with Trolox (0–250 μmol/L) was used, and the results were expressed as mmol TE/100 g DW.

#### 2.4.6. Antioxidant Activity by Silver Nanoparticle

To determine the antioxidant activity, the silver nanoparticle method was used according to Pintea et al. [38]. This is a simple spectrophotometric method based on the reduction of silver ion Ag+ (a solution of silver nanoparticles stabilized with sodium citrate and starch) to metallic silver Ag^0^ nanoparticles, in the presence of antioxidants–polyphenols extracted from pumpkin powder. The absorption of the samples is read at λ = 423 nm, against the blank [39].

The pumpkin powder extract was obtained in the following way: 0.5 g of pumpkin powder was extracted by shaking the sample for 1 min at 2000 rpm in a VX-200 vortex mixer (Labnet, Edison, NJ, USA) with 5 mL of methanol. The extraction was carried out using the ultrasound-assisted method (ISOLAB Laborgeräte GmbH, Germany) at a frequency of 37 kHz for 30 min; then, the mixture was centrifuged at 3000 rpm for 10 min and filtered. For quantification, we used the gallic acid calibration curve (0–0.05 g/L, y = 20.646x + 0.0054, R^2^ = 0.9963). The results were expressed in gallic acid equivalents per 100 g dry weight of the product (mg GAE/100 g DW).

### 2.5. Antimicrobial Activity

#### 2.5.1. Preparation of Inoculum

Antimicrobial properties of extracts (pumpkin powders were tested against Gram-positive bacteria (*Staphylococcus aureus* ATCC 25923, *Bacillus cereus* ATCC 11778), Gram-negative bacteria (*Escherichia coli* ATCC 19433, *Salmonella Abony* ATCC *BAA-2162, Pseudomonas aeruginosa* ATCC 27853) and yeast (*Candida albicans* ATCC 10231). The strains used were purchased from the American Type Culture Collection (ATCC). Gram-positive and Gram-negative microorganisms were precultured in Müeller–Hinton broth at 37 °C for 18–24 h. Subsequently, a suspension was prepared from each microbial strain with a concentration of 10^8^ cells/mL according to the McFarland standard of 0.5 [40]. Candida yeasts were cultivated for 48 h on potato dextrose agar at 37 °C, and after that the inoculum was prepared with a final concentration of 10^6^ cells/mL [41].

#### 2.5.2. Antimicrobial Activity Detection Using the Agar Well Diffusion Method

Antimicrobial activity was determined using the agar well diffusion method, as displayed by Ben Hsouna et al. [42]. Wells with 8 mm diameter were made on Müeller–Hinton agar plates using a sterile metallic cylinder. The previously prepared inoculum was spread uniformly on the surface of the agar plates using a sterile swab. In each well, 100 μL of dissolved powder (125 mg/mL dimethylsulfoxide (DMSO)) was introduced. The plates were kept at +4 °C for 2 h to ensure the diffusion of the extracts in the agar [43], subsequently being incubated at 37 °C for 24 h (bacteria) and 48 h (yeasts). The measurement of the total zone of inhibition (including the diameter of the wells) after the incubation period allowed for the detection of antimicrobial activity. DMSO was used as a negative control. All tests were performed in triplicate.

#### 2.5.3. Determination of MIC, MBC and MFC Using the Microdilution Well Method

The goal of our study was to determine whether the etiologic agent is resistant or sensitive to the natural antimicrobial agents being tested. The minimum inhibitory concentration (MIC) values were determined using the method described by Gulluce et al. [44]. In a sterile 96-well microtiter plate with a total volume of 200 µL per well, initially or after dispensing 100 µL of Müeller–Hinton peptone broth. Afterwards, we performed twofold serial dilutions of pumpkin powder in the range of 0.25–150 mg/mL. A microbial suspension of 10μL was added to each well with a final inoculum concentration of 10^8^ cells/mL for bacteria and 10^6^ cells/mL for yeasts.

The contents were homogenized; then, the plates were covered with sterile lids and incubated for 24 h at 37 °C (bacterial strains) and for 48 h at 37 °C (fungal strains). The lowest concentration of powder inhibiting the growth of microorganisms was established—MIC.

The minimum bactericidal concentration (MBC) values were interpreted as the smallest amount of powder inhibiting the visible growth of the microorganisms. For determination of MBC, each dilution was subcultured on Müeller–Hinton agar plates with subsequent incubation of the plates and interpretation of the results. The MBC value is determined by the lowest concentration of the tested compound that reduces the number of colonies on the plate by up to 99.9% [45].

The minimum fungicidal concentration (MFC) was determined by serially subculturing 10 μL in potato dextrose agar plates and incubating for 48 h at 37 °C. MFC represented the lowest concentration that inhibited yeast growth [46].

All assays were caried out in triplicate and the results were expressed as a mean value ± standard deviation (SD).

### 2.6. Preparation and Characterization of Shortbread Cookies with Pumpkin Powder

#### 2.6.1. Preparation Shortbread Cookies with Pumpkin Powder

Samples of shortbread dough were made using 5% to 20% yellow pumpkin powder (5% YPP, 10% YPP, 15% YPP and 20% YPP) to determine its effects on the sensory and physicochemical quality of the shortbread cookies. YPP was added directly to wheat flour. For the control, a sample was prepared without the addition of YPP.

Premium quality wheat flour (moisture content: 13.7%, ash content 0.6%), powdered sugar (average crystal size: 120 μm), butter (moisture content: 16.9%, fat content: 82.5%) and potato starch (moisture content—18%) were used to make shortbread cookies.

Soft butter and powdered sugar were mixed for 6 ± 1 min. Afterwards, wheat flour with potato starch, and with/without YPP was poured in slowly while stirring. The dough, kneaded for 5–10 min, was left to rest for 30 min at 20 ± 1 °C. The dough was rolled on lightly floured surface to an even thickness of 5 mm. The biscuits were stamped out using a round cookie cutter with a diameter of 60 mm, keeping cuts close together. The stamped biscuits (20 ± 1 g) were baked in an electric convection oven (EASY EV-UME604-LS, Luxstahl, PiggioTorriana, Italy) at 180 ± 2 °C for 12 ± 1 min until cooked. After baking, the biscuits were packed and stored in a dry place at room temperature.

The quantities of each ingredient used in the preparation are presented in Table 2.

#### 2.6.2. Texture Profile Analysis of Dough and Shortbread Cookies

A Stable Micro Systems TA.HD plus C analyzer, UK was used for texture profile analysis of dough. The textural properties (hardness, cohesiveness, gumminess, resilience, adhesiveness) of the dough samples were determined with a 40 mm cylindrical probe through the double compression test, using P/75 stainless plate and respecting the following parameters: pretest speed—100 m/s; test speed—5 m/s; posttest speed—5 m/s; cell load—5 kg [47].

The texture parameters of shortbread cookies were made using a texture analyzer (Stable Micro Systems TA. HD plus C, Godalming, UK) equipped with a 5 kg load cell. The breaking force or hardness (g) of cookie samples was performed using the three-point bend test with an HDP/3PB knife-edge probe. The biscuit samples were placed on base beams with a distance of 4 cm between the two beams. The analyzer was set to a return-to-start cycle with the following parameters: pretest speed 1 mm/sec; test speed 2 mm/s; posttest speed 10 mm/s; trigger force—5 g; distance—5 mm [48].

#### 2.6.3. Sensory Analysis of Shortbread Cookies

The sensory properties of the shortbread cookies samples were analyzed according to ISO 6658:2017 [49]. Appearance, taste, odor, color and consistency of five cookie samples were evaluated by a trained panel comprising academic staff and food industry experts with necessary knowledge and experience consisting of 11 persons. Sensory analysis was performed in individual booths at room temperature using white light. The panel developed a list of attributes and evaluated the samples using a 5-point method: 5 = Like extremely. Exceptional, ideal qualities, 4 = Like. Suitable qualities, 3 = Slightly. With slight defects, 2 = Dislike. With obvious defects, 1 = Dislike extremely. With strongly pronounced defects, 0 = Altered, with large changes. Each sample was tested in duplicate in sensory laboratories that met the requirements of ISO 8589 [50]. The cookies were served in plastic containers with lids, coded with three-digit random numbers, and presented to the raters in randomized order. A rest period of 2–3 min between assessments was provided. Warm water was used to rinse the mouth.

#### 2.6.4. Physicochemical Analysis of Shortbread Cookies

The moisture content (MC) of the shortbread cookies was determined according to the AACC standard (AACC, Method 44-15.02) [51]. The water swelling of shortbread cookies was determined according to [18]. The method is based on the measurement of the mass difference in the products immersed in water at a temperature of 20 ± 1 °C for 2 min, as recommended for flour confectionery products. The water activity (a_w_) of the baked cookies was measured at room temperature (25 ± 1 °C) using an electronic dew-point water activity meter LabMaster (Novasina AG, CH-8853 Lachen, Switzerland) [52]. Measurement of a_w_ was carried out until the value was concurrent.

### 2.7. Statistical Analysis

The test results are presented in this paper as mean values ± standard error of the mean for three parallel measurements. The Microsoft Office Excel 2007 program (Microsoft, Redmond, WA, USA) was used for statistical processing. One-way analysis of variance (ANOVA) was performed according to the Tukey test, with a significance level of *p* ≤ 0.05. Staturphics software Centurion XVI 16.1.17 (Statgraphics Technologies, Inc., The Plains, VA, USA) was used.

## 3. Results and Discussion

### 3.1. Characterization of Pumpkin Fresh Pulp and Powder

The physicochemical indicators and color parameters determined for fresh pumpkin pulp are presented in Table 3.

Moisture values for butternut pumpkin samples (89.6%) were somewhat lower compared to the MC of fresh pumpkin pulp, which, according to Adubofuor et al. and See et al., were 92.24–95.66% [53,54]. These values correlate with the data recorded for pumpkin pulp of the cylindrical variety *Cucurbita moschata*—89.5–89.65% [55,56]. The rather high MC of pumpkin fruit pulp indicates the susceptibility of the fruit to microorganisms and spoilage [57].

There are differences in how fruits and vegetables are categorized based on pH. Pumpkin fruit is generally classified as a medium-acidic product with a pH range of 4.99–5.50 [58]. The values obtained for the butternut variety study were above the range 4.90–5.50 reported for pumpkin fruits [58,59]. According to the values obtained in this study, the fruits are within the pH range of 5.0 and above the pH range established for low-acid products [60]. The pH value of the fresh pulp was 6.01 (Table 3). Malkanthi et al. [26] recorded a pH of 5.75 for dry pumpkin powder (10 g powder in 100 mL water), the acidity of which acidity was due to the dry form of the sample. Over the course of the study, it was found that the content of dry matter in the studied variety of pumpkin was 8.97%, which agrees well with the result of organoleptic evaluation of the pulp. It should be noted that the studied pumpkin variety contains an insignificant number of acids (this fact explains the bland flavor of the pulp).

For the fresh pulp, the FC, PC, TFC and AC, expressed in g/100 g DW, were determined as 2.13, 8.42, 7.53 and 2.98, respectively (Table 3). The values recorded by Kim et al. [29] were 4.20, 11.3, 10.88 and 10.53 for lipid, protein, fiber and ash of pumpkin flesh, expressed in g/kg raw weight. The AC in pulp of the studied variety (2.98%) was significantly higher than that of pumpkin fruit pulp reported in the scientific works [53,54] and compares well with the rates of 2.14–4.26% obtained in the scientific works [55,61]. Differences in AC may be due to varietal and geographical differences in pumpkin fruit cultivation [62].

In the pumpkin pulp drying process, at the temperature of 60 ± 2 °C, the physicochemical parameters determined for the powder did not change significantly (Table 3). Other authors [20] established that hot-air drying at temperatures of 70 °C ensures very good quality of pumpkin powder and increased antioxidant potential. The MC (6.88%) was lower than in a previous study [63] (10.14%), but was within the safety limit, as Bothast et al. [64] noted that pumpkin powder with more than 14% moisture is susceptible to fungus and mold growth. In the pumpkin powder, the titratable acidity was 1.05%, expressed in citric acid. These values correlate with Dhiman et al.—1.03% [65].

In pumpkin powder, higher values (g/100 g DW) for lipid, protein, fiber and ash content of 5.74, 11.68, 9.69 and 4.07, respectively, were recorded. On the other hand, ash (4.07%) in pumpkin powder was reported by Ptitchkina et al. [66]. This phenomenon was probably due to the processes of splitting macromolecular complexes during the procurement of the dry powder, which led to the release of lipids, proteins, and other components from the vegetable product matrix.

See et al. [54] determined that in fresh pumpkin, the contents of moisture, fat, protein, ash, fiber and carbohydrate were 92.24, 0.15, 0.98, 0.76, 0.56 and 5.31%, respectively, whereas moisture, fat, protein, ash, fiber and carbohydrate in pumpkin powder were 10.96, 0.80, 9.65, 5.37, 0.81 and 72.41%, respectively. Bhat et al. [67] determined a lower β-carotene content in fresh pumpkin (2.44 mg/100 g) compared to that in dried powder (7.30 mg/100 g).

The physicochemical parameters of the pumpkin powder also fall within the limits of the values obtained by several researchers (Table 3). Malkanthi et al. [27] determined the following contents (g/100 g) of moisture, protein, carbohydrates and ash in pumpkin flesh powder: 14.8, 8.95, 67.00 and 5.56, respectively. Jabeen et al. [68] determined 9.9 g moisture, 2.3 g fat, 3.07 g protein, and a high amount of fiber (11.46 g) and ash (15.98 g/100 g) in pumpkin flour.

Nor et al. [69] reported that pumpkin powder contained 7.10%, 3.10%, 1.80%, 5.70%, and 82.30% of protein, fat, moisture, ash, and carbohydrates, respectively. Badr et al. [14] determined the proximate composition of pumpkin flesh powder; the values for ash, fat, fiber, moisture, protein and carbohydrates (g/100 g DW) were 6.64, 0.18, 11.25, 18.03, 15.50 and 48.40, respectively.

The values of the chromatic parameters L*, a* and b* were analyzed in pumpkin pulp and powder (Table 3). The L* values show that the pumpkin powder (69.33) was brighter than the pulp (59.84). The a* value of fresh pumpkin was higher (15.04) than powder (6.67), indicating more redness in fresh pumpkin. The b* value of fresh pumpkin (69.16) was also higher than for pumpkin powder (43.1), demonstrating a greater presence of yellow pigments. This transformation can be explained by the isomerization of trans carotenes into various cis isomers, recognized as one of the mechanisms for the degradation of carotenoids in food [70]. Dehydration in hot air exposed the color pigments to the action of oxygen, and the activity of the enzymes lipoxygenase and peroxidase, responsible for oxidative degradation, also contribute to the reduction in pigment content [71].

### 3.2. Chemical Composition and Antioxidant Activity of the Pumpkin Powder

The research carried out in the present study showed that the biological value of pumpkin powder increased due to the qualitative and quantitative content of antioxidants determined using UV-VIS spectroscopy and RP-HPLC chromatography (Table 4).

The values determined for TPC of 363.15 mg GAE/100 g DW and TFC 77.83 mg QE/100 g DW in the acidified methanolic extracts (85:15, *v*/*v*, MeOH:HCl) of pumpkin powder variety butternut squash were higher, and the carotenoid content of 27.76 mg/100 g DW was lower (Table 4) compared to the values determined by several researchers. Thus, Hussain et al. [4] obtained powder from pumpkin flesh, dried first in the sun, then in a conventional hot-air oven, at 60℃. For the 80% aqueous methanolic extract, these authors recorded lower values for TPC (134.59 mg GAE/100 g powder) and TFC (77.11 mg CE/100 g, catechin equivalent), but determined higher TCC content (35.2 mg/100 g) using the HPLC method.

The content of biologically active substances in plant powders depends on several factors [8,9,29], including the method applied to obtain the extracts [72]. Thus, Asif et al. [21], for methanolic extracts of 65%, 80%, 99.9% pumpkin puree, determined TPCs of 6.78, 5.15, 4.31 mg GAE/100 g and TFCs of 0.72, 0.65, 0.51 mg GAE/100 g, respectively.

Wang et al. [73] noted that the carotenoid content in *Cucurbita maxima* dried slices extracted with 100% acetone was not influenced by the drying method. TCC determined in hot-air-dried samples was 67.6 mg/100 g, and in freeze-dried samples 63.7 mg/100 g.

Research showed that the TPC, TFC and TCC determined in this study provide an excellent antioxidant potential of 161.52 mmol TE/100 g DW for pumpkin powder applied in shortcake manufacturing. Other research showed an antioxidant activity of pumpkin pulp powder of 0.53 mmol AAE/100 g (ascorbic acid equivalents), a TPC of 192 mg GAE/100 and a β-carotene content of 32.87 mg/100 g DW [27].

In pumpkin puree, it was found to be 23.6 mg/100 g of phenol content and 0.102 mmol TE/100 g antioxidant activity [74]. Another experiment revealed that the phenolic content of pumpkin pulp powder ranged from 159.69 to 35.94 mg GAE/L, whereas the antioxidant activity ranged from 0.284 to 0.135 mmol AAE/L [75].

In the present study, the antioxidant activity of phenolic compounds from pumpkin powder against the formation of silver nanoparticles was evaluated. The plasmonic response of silver nanoparticles reduced with phenolic compounds was evaluated (558.71 mg GAE/100 g DW) and correlated with the antioxidant capacity of gallic acid. The relationship between the antioxidant capacity of phenolic acids and the corresponding optical response of plasmons can be used as an innovative antioxidant detection test for samples rich in phenolic compounds. Nanoparticle-based antioxidant assays have been shown to be sensitive, and rapid methods for determining the antioxidant capacity of phenolic compounds correlate well with classical antioxidant assays such as DPPH and ABTS [39].

**Figure 1:**
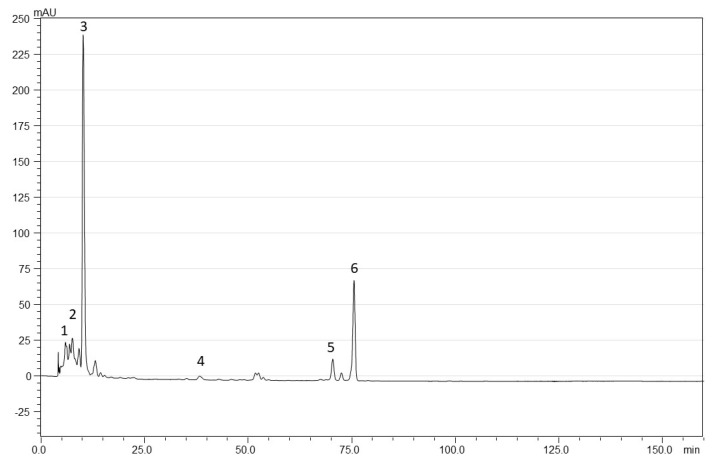
RP-HPLC chromatograms of saponified carotenoid extracts of butternut squash powder: 1-Luteoxanthin; 2-Violaxanthin; 3-Lutein; 4-*β*-cryptoxanthin; 5-*Cis-β*-carotene; 6-all-*trans-β*-carotene.

According to the literature review, the TCC value is higher if minor carotenoids and their esterified forms are considered. The main carotenoids identified in pumpkin were *β*-carotene, *α*-carotene, lutein and zeaxanthin. *β*-carotene, the precursor of vitamin A, is the major carotenoid in pumpkin species [76]. Table 4 and Figure 1 show the profile of saponified carotenoids, identified in butternut squash powder, of which lutein has the highest content (2.19 mg/100 g DW), followed by all-*trans-β*-carotene (0.66 mg/100 g DW). Compared to other bibliographic results [76], the lutein content recorded in our research was modest, although it was mentioned in the literature that in three pumpkin species, the lutein concentration varied from 0 to 17 mg/100 g [77].

To determine the concentration of *α-* and *β*-carotenes and their isomers, Garvalho et al. [78] applied HPLC to the pulp of two local species (A and B) of fresh *Cucurbita moschata*. The results showed that (all-E)-*β*-carotene was the most abundant in both samples, with values of 244.22 and 141.95 µg/g in samples A and B, respectively. The values for (9Z)-*β*-carotene were 2.34 µg/g (A) and 0.97 µg/g (B), and those for (13Z)-*β*-carotene were 3.67 (A) and 1.84 µg/g (B).

Kim et al. [29], using the HPLC method, determined the following concentrations of carotenoids in *Cucurbita maxima* pumpkin pulp: *α*-tocopherol—2.31; *β*-carotene—17.04; *β*-cryptoxanthin—0.65 mg/kg raw weight. In our research, the content of *β*-cryptoxanthin was lower (0.27 mg/100 g DW).

The lower concentration of carotenoids in saponified form, identified in our research, was probably influenced by the extraction method and the saponification process of carotenoids.

### 3.3. Antibacterial Activity of the Pumpkin Powder

In this study, the antimicrobial activity of pumpkin powder against *Staphylococcus aureus* ATCC 25923, *Bacillus cereus* ATCC 11778, *Escherichia coli* ATCC 19433, *Salmonella Abony* ATCC BAA-2162, *Pseudomonas aeruginosa* ATCC 27853 and *Candida albicans* ATCC 10231 was evaluated (Table 5). Initially, the antimicrobial activity of the pumpkin powder was qualitatively assessed by the presence or absence of microorganism growth inhibition zones. The obtained results demonstrated that the pumpkin powder has a bactericidal effect both on Gram-positive and Gram-negative bacteria, as well as on *Candida* yeasts.

Pumpkin powder demonstrated antibacterial activity with the maximum zone of inhibition for *Candida albicans* (19.0 mm). For *Pseudomonas aeruginosa*, the zone of inhibition was 14.0 mm, and for *Bacillus cereus* and *Escherichia coli* it was 12.0 mm. The smallest zone of inhibition was recorded for the species *Salmonella Abony* (10.0 mm).

The results of the study on the MIC and MBC values of the pumpkin powder are presented in Table 5. The MIC for *Bacillus cereus* and *Candida albicans* strains was 18.75 mg/mL, and for *Staphylococcus aureus, Escherichia coli* and *Pseudomonas aeruginosa* it was 37.5 mg/mL. Antimicrobial activity in higher concentrations was recorded on strains of *Salmonella Abony*—75.0 mg/mL. Pumpkin powder also demonstrated a bactericidal effect at a concentration of 37.5 mg/mL on *Bacillus cereus* and *Candida albicans*. The MBC for strains of *Staphylococcus aureus*, *Escherichia coli* and *Pseudomonas aeruginosa* was 75.0 mg/mL, and for *Salmonella Abony* it was150.0 mg/mL. The antimicrobial effect of pumpkin powder is due to the high concentrations of polyphenols (363.15 mg GAE/100 g DW) and flavonoids (77.83 mg QE/100 g DW) that it contains. These compounds are responsible for the antimicrobial activity due to the polar isopropyl functionality of the phenolic components. Polyphenols and flavonoids from pumpkin powder react with cellular components of microorganisms and lead to leakage of nucleotides and proteinaceous material into extracellular areas [10].

Asif et al. conducted research to investigate the antibacterial activity of 65%, 80% and 99.9% methanolic extracts of pumpkin peel and flesh against *Pasteurella multocida, Escherichia coli, Staphylococcus aureus* and *Bacillus subtilis.* The diameter of inhibition zone of methanolic extracts of pumpkin peel and flesh against *Pasteurella multocida* was greater than 15 mm, while the zone of inhibition against three other bacterial species was in the range of 10 to 15 mm [21].

Dissanayake et al. determined the antimicrobial effect of pumpkin peel, seed and leaf extracts using acetone, methanol and ethyl acetate solvents on *Staphylococcus aureus*, *Bacillus subtilis* and *Escherichia coli* bacteria. Methanolic extract of pumpkin peel showed the highest activity against *Staphylococcus aureus* with zones of inhibition of 7.58 mm and 4.83 mm for *Bacillus subtilis*, and no antimicrobial activity was found for *Escherichia coli.* On the other hand, methanol extracts of pumpkin seeds showed no antimicrobial effect, but ethyl acetate extract of pumpkin seeds showed an inhibition zone of 6.61 mm against *Staphylococcus aureus* [79].

Tyan et al. [80] evaluated the antimicrobial activity of pumpkin extracts on seven microbial strains and proved that *Cucurbita moschata* extracts possess antimicrobial activity against all strains taken in the study.

Chonoko and Rufai [81] conducted research on the antimicrobial activity of pumpkin and reported a zone of inhibition in the range of 7–10 mm of both ethanolic and methanolic extracts of pumpkin peel against bacterial species *Staphylococcus aureus* and with the zone of inhibition of 6–12 mm against *Salmonella typhi*.

Badr et al. [14] found that pumpkin seed oil exhibited strong antifungal activities against the fungal species *Saccharomyces cerevisiae*. The antimicrobial effect exerted by pumpkin powder is probably due to saponins, tannins, flavonoids, alkaloids and steroids present in pumpkin [81].

Hussain et al. [10] tested the antimicrobial activity of three types of pumpkin methanolic extracts against various species of bacteria and fungi. Significantly different values were obtained for the antibacterial activity of pumpkin peel, flesh and seed extracts against the microbial species taken in the research. Against *Salmonella typhi*, all three types of extracts showed significant antibacterial activity. Against *Escherichia coli* and *Bacillus subtilis* species, pumpkin flesh extract showed a larger zone of inhibition compared to peel and seed extracts. Against *Staphylococcus aureus*, pumpkin peel, flesh and seed extracts showed nonsignificant activity.

The antimycotic activity of these extracts was also demonstrated. The highest activity against the species *Candida albicans* was demonstrated by the methanolic extract from seeds (9.34 mm), followed by the extract from the peel (8.69 mm) and flesh (7.80 mm) of pumpkin [10].

### 3.4. Characterization of Shortbread Cookies

Sensory evaluation of the products showed that the highest average score was given to 15% YPP (4.9 points), 20% PSC (4.55 points), and control samples (4.59 points) (Table 6). The highest rating for the consistency of cookies was obtained with the use of YPP 15%—4.87 points. Replacing 20% of wheat flour with YPP slightly reduced the score to 4.48 points. The control sample and cookies with the addition of 5–10% YPP had a crumbly consistency. The use of 15–20% pumpkin powder contributed to firmer cookie consistency. According to Seyhun et al. [82], the firmer cookie consistency with 15–20% YPP can be attributed to amylose and amylopectin recrystallization, the formation of complexes between starch and proteins, and to redistribution of water between the components of the product, as well as other events that may occur in this type of cookie.

The highest rating for the taste of cookies was obtained in the samples with the addition of 10 and 15% YPP—4.94 and 4.99 points, respectively. The use of 15% YPP provided an odor at the highest level of 4.84 points. The use of a higher amount of pumpkin powder slightly reduced this indicator to 4.45 points. Products with high YPP content (15–20%) had a pronounced nutty flavor and odor.

The resulting shortbread cookies have a particular orange yellow color, which is typical of the substitute ingredient, which is YPP. Assessed simultaneously regarding all attributes, the result that received the highest rating was cookies with 15% pumpkin powder, with a score of 4.83. The cookies with high concentration of YPP (15–20%) had a dark orange color, which is the result of a more intensive Maillard reaction, which depends on the content of sugars and proteins [83,84,85]. It should be noted that all products had an appropriate shape, without bloating and dents, with a smooth surface. Taste and odor were typical of the ingredients included in the product, without extraneous taste and odor.

Thus, the introduction of a natural component of raw plant materials—YPP—into the developed biscuits recipe significantly improved organoleptic parameters, in particular, the taste, odor and color of the products, which was noted by all tasters. In addition, the biscuit samples obtained using the developed formulation had a better structure and appearance, being denser and moister in texture compared to the control sample. The introduction of YPP was found to compensate for the lack of flavor compounds compared to the control. However, the addition of 20% YPP has a slight negative effect on taste and odor (more pungent nutty taste). Overall, the addition of YPP in an amount not exceeding 15% improves the overall consumer experience and has practical applications in the development of a better and more presentable product.

The MC of cookies increased linearly from 3.37% to 3.87% with an increase in concentration of YPP, which can be explained by the biochemical composition of the YPP, in particular, a fairly high content of proteins and dietary fiber, which account for the high water-binding capacity of pumpkin powder, which retained more moisture in the finished products. The results for moisture content of the shortbread cookies were similar to the results obtained by Mishra and Kalpana [86], who incorporated potato flour in preparation of the cookies, similar to the results obtained by Dziki et al. [87], who incorporated oat husk in preparation of the cookies. The MC obtained for shortbread cookies with YPP compares well with the rates obtained in the scientific works [88,89].

The swelling in water of the cookies decreased as the amount of YPP was increased. This is due to the fact that the dough with increasing replacement of top-grade wheat flour with YPP becomes more plastic, which further allows for obtaining a more loosened and brittle cookie structure. It was found that with increasing quantity of YPP in the recipe of shortbread cookies, wetness of the products decreases by 9.1% upon introduction of 15% of YPP from the weight of flour and by 11.9% at 20%. This can be explained by the presence of pectin substances in pumpkin powder, which retain a significant amount of water.

Cookie a_w_ is very important to predict its stability and safety regarding microbial growth and lipid oxidation rates. The a_w_ of all measured cookies were in the range of 0.177 to 0.199 (Table 6). According to the reference, the a_w_ for food spoilage by bacteria, yeasts and molds are 0.90, 0.85–0.88 and 0.80, respectively [90]. The a_w_ significantly influences the shelf life of the product. The higher the a_w_ value, the more likely the development of microorganisms and product decomposition processes. a_w_ values lower than 0.60 prevent microbial spoilage and exhibit microbiological stability [91].

The use of pumpkin powder led to a decrease in the hardness of the cookie dough. Pumpkin powder had a different chemical composition from wheat flour in terms of fat, dietary fiber and gluten content. A drastic decrease of about two times was observed between the CS and the sample where the wheat flour was substituted with only 5% YPP. This dependence was also observed for the other samples, respectively, with a decrease in hardness by 65–67%. The composite dough of wheat flour and pumpkin powder had much lower hardness compared to the CS, possibly due to higher fat content in the pumpkin powder. Similar results have been obtained by other authors [92] in the manufacture of cuts with the addition of pumpkin flour. Also, Wongsagonsup et al. [93] obtained decreasing results for bread dough hardness when made from a mixture of wheat flour and pumpkin flour. This decrease in hardness results was explained by the weakening of the gluten matrix, since pumpkin powder does not contain gluten.

The respective changes in the dough structure also influenced other texture parameters; namely, for adhesiveness, decreasing values were recorded for all the dough samples with the addition of pumpkin powder—a significant decrease of over 55% for 5% YPP compared to the CS. Resilience also showed decreasing values, with a maximum of 13.7% for the 20% YPP and a minimum of 8% for 5% YPP. Cohesiveness, which represents the internal links that hold together the structure of composite flour gels [94], remains practically unchanged for all samples; the amount of pumpkin powder did not influence the results of this parameter. The evolution of resilience and cohesiveness may be due to the weakening of the dough matrix initiated by the increase in dietary fiber content, which also influences the ability of the dough to retain gases [95].

Considering that gumminess is related to the evolution of hardness and cohesiveness [96], this parameter also showed decreasing values. The gumminess of the dough samples with the addition of pumpkin powder compared to the control sample decreased by almost 50% for the 5% YPP, by 61.8% for the 10% YPP, 64.2% for the 15% YPP, and by almost 65% for the 20% YPP.

Similar results were obtained by Aljahani [94] for composite gels from wheat flour and pumpkin powder (5%, 10% and 15%), a decrease in firmness and gumminess of a maximum of 23.3% and, respectively, 25% for nonessential cohesiveness changes.

Hardness is a quality indicator specific to cookies. According to the data presented in Table 6, the values for hardness increased compared to the CS. Increasing the amount of pumpkin powder in the shortbread cookie recipe led to an increase in this texture parameter, by approximately 36% for the 5% YPP, 45% for the 10% YPP, 48% for the 15% YPP and 50% for the 20% YPP. The increase in shortbread cookie hardness values was due to the weakening of the gluten network, due to the addition of pumpkin powder. Liubych et al. [97] showed that the use of pumpkin powder in the manufacture of cookies led to an increase in breaking strength: there was an increase from 1.05 kg for the control sample to 1.4–4.0 kg for samples with 5–20% pumpkin flour. The authors argued that the increase in breaking strength values of the composite cookies was due to their small volume as well as the reduction in the degree of swelling due to the reduced absorption capacity of the pumpkin powder.

Table 6 shows the values of the color parameters L*, a* and b* for the core and crust of the shortbread cookies. It was found that in both cases, for all cookie samples (CS and YPP), the brightness values (L*) were over 50 and were in the clear zone [98]. It was found that the L* of the cookies showed a decreasing trend with the increase in the level of substitution of flour with pumpkin powder. Lower values of L* indicated that the cookies were more closed at higher levels of substitution compared to the CS. This effect was caused by the presence of natural pigments, such as carotenoids, which are naturally found in pumpkin powder [76,77]. Also, crust L* values are lower for YPP due to the result of browning and Maillard reactions, which depend on the content of reducing sugars and proteins on the sample surface [85].

The values of the parameters a* and b* in both cases (core and crust) of CS and YPP were positive, demonstrating the predominance of red color over green and a strong predominance of yellow coloration, in disfavor of the blue, respectively. The resulting color of the cookies was a dark yellow. It was also found that the values of parameters a* and b* in the samples with pumpkin powder were higher than in the case of the CS. This is probably due to the natural coloring pigments, carotenoids, in the pumpkin powder. In general, the a* and b* parameter values of the shortbread cookies crust were higher compared to their core. YPP samples contained more sugar, which facilitates browning and Maillard reactions.

ΔE* represents a dimensionless parameter, resulting from the combination of the L*, a* and b* values of the pairs of samples, which indicates whether or not there are differences in the colors perceived by the human eye, depending on the specific sensory thresholds [99]. Lo Faro et al. showed the difference between colors: if ΔE < 0.2—an imperceptible difference; if 0.2 < ΔE < 0.5—a very small difference; if 0.5 < ΔE < 1.5—a small difference; if 2 < ΔE < 3—a barely distinguishable difference; if 3 < ΔE < 6—a very distinguishable difference; if 6 < ΔE < 12—a large color difference; if ΔE > 12—completely different colors. [99]. The CS was analyzed for shortbread cookies with different substitutions of pumpkin powder. The values of ΔE* were found to be between 6 < ΔE* < 12, a large color difference, and ΔE* > 12, completely different colors [100]. The ΔE* of the cookie crust and core was also influenced by the presence of pumpkin powder, and the ΔE* values increased with increasing replacement of pumpkin powder in the cookies. The pumpkin composition significantly influenced color differences, since the baking conditions were the same for all samples obtained.

## 4. Conclusions

In the process of drying pumpkin pulp at 60 ± 2 °C, the physicochemical parameters did not change significantly. Significant amounts for lipid, protein, fiber and mineral content, respectively, of 5.47, 11.68, 9.69 and 4.07 g/100 g DW were recorded in pumpkin powder. The chromatic parameters L*, a* and b* showed that the pumpkin powder was brighter than the pulp, with the greater presence of yellow pigments.

The content of polyphenols, flavonoids, total and individual carotenoids of the pumpkin powder was determined. It was found to be a rich source of bioactive compounds, with an antioxidant potential of 161.52 mmol TE/100 g DW. The profile of saponified carotenoids in pumpkin powder showed that lutein has the highest content (2.19 mg/100 g DW), followed by all-trans-β-carotene (0.66 mg/100 g DW).

Pumpkin powder shows antimicrobial activity against both Gram-positive (*Staphylococcus aureus*, *Bacillus cereus*) and Gram-negative (*Escherichia coli*, *Salmonella Abony* and *Pseudomonas aeruginosa*) bacteria. High antifungal activity against *Candida albicans* species was detected.

Sensory, physicochemical and texture parameters and color indicators of pumpkin shortbread cookies with 5–20% YPP added, in relation to the CS, were analyzed. Sensory evaluation showed that the highest average score was achieved by the 15% YPP sample. The use of 15–20% YPP contributed to firmer cookie consistency due to the formation of complexes between starch and proteins.

The dough with increasing replacement of top-grade wheat flour with YPP became more plastic, which allowed for obtaining a more brittle cookie structure. As the amount of pumpkin powder in the cookie recipe was increased, a constant increase in the breaking strength values of the cookies was attested due to the weakening of the gluten network and the reduced absorption capacity of the pumpkin powder.

The brightness of the cookies showed a decreasing trend with the level of replacement of wheat flour with pumpkin powder; the resulting color of the cookies was dark yellow. The values of parameters a* and b* in the samples with pumpkin powder were higher than in the case of CS due to the natural coloring pigments, carotenoids. YPP samples had higher sugar content, which facilitated browning and Maillard reactions. The difference in colors perceived by the human eye of the cookie’s crust and core increased with the replacement of pumpkin powder in the cookies.

Research results showed that pumpkin powder considerably improves the sensory and textural characteristics of biscuits and is recommended for extending the range of high-quality and food-safe flour products.

## Figures and Tables

**Table 1:** Spectral characteristics and retention times of carotenoids identified using RP-HPLC analysis.

Compound	Retention Time (min)	Max Absorption (nm)
Luteoxanthin	7.498	401, 420, 448
Violaxanthin	9.122	401, 420, 444
Lutein	10.121	421, 445, 473
*β*-cryptoxanthin	38.246	428, 451, 476
*Cis-β*-carotene	70.383	424, 446, 472
All-*trans-β*-carotene	75.511	421, 452, 478

**Table 2:** Formulation of the shortbread cookies.

Ingredients	CS	5% YPP	10% YPP	15% YPP	20% YPP
Wheat flour, g	100	95	90	85	80
YPP, g	0	5	10	15	20
Powdered sugar, g	40	40	40	40	40
Butter, g	80	80	80	80	80
Potato starch, g	13	13	13	13	13

CS = 100% wheat flour; 5% YPP = 5% yellow pumpkin powder; 10% YPP = 10% yellow pumpkin powder; 15% YPP = 15% yellow pumpkin powder; 20% YPP = 20% yellow pumpkin powder.

**Table 3:** Physicochemical indicators and color parameters (CIELab) of the pumpkin fresh pulp and powder.

Parameters	Fresh Pumpkin Pulp	Pumpkin Powder
Physicochemical Parameters		
Moisture content, g/100 g	89.6 ± 0.03	6.88 ± 0.05
pH	6.01 ± 0.30	n/d
Titratable acidity, % expressed in citric acid	n/d	1.05 ± 0.01
Soluble solids content, g/100 g	8.97 ± 0.06	n/d
Fat content, g/100 g DW	2.13 ± 0.10	5.74 ± 0.21
Protein content, g/100 g DW	8.42 ± 0.21	11.68 ± 0.11
Total fiber content, g/100 g DW	7.53 ± 0.17	9.69 ± 0.35
Ash content, g/100 g DW	2.98 ± 0.01	4.07 ± 0.14
CIELab Chromatic Parameters		
L*	59.84 ± 0.21	69.33 ± 0.13
a*	15.04 ± 0.06	6.67 ± 0.08
b*	69.16 ± 0.11	43.1 ± 0.05

Values represent means of three replicated trials, ± standard deviation; n/d = not determined; L* = luminosity; a* = red/green component; b* = yellow/blue component.

**Table 4:** The content of total polyphenols, flavonoids, total and individual carotenoids, and the antioxidant activity of pumpkin powder used for experiments.

Indices	Mean Value ± SD
Total polyphenols, mg GAE/100 g DW	363.15 ± 16.8
Total flavonoids, mg QE/100 g DW	77.83 ± 9.01
Total carotenoids, mg /100 g DW	27.76 ± 5.01
Luteoxanthin, mg/100 g DW	0.50 ± 0.16
Violaxanthin, mg/100 g DW	0.45 ± 0.07
Lutein, mg/100 g DW	2.19 ± 0.11
*β*-cryptoxanthin, mg/100 g DW	0.27 ± 0.04
Cis-*β*-carotene, mg/100 g DW	0.26 ± 0.09
All-trans-*β*-carotene, mg/100 g DW	0.66 ± 0.13
Antioxidant activity (DPPH), mmol TE/100 g DW	161.52 ± 21.6
Antioxidant activity by silver nanoparticles, mg GAE/100 g DW	558.71 ± 11.3

Values represent means of three replicated trials, ± standard deviation. DPPH = 2,2 diphenyl-1-picrylhydrazyl-hydrate.

**Table 5:** Antimicrobial activity of pumpkin powder.

Test Strains	Zone of Inhibition, mm	Powder Concentration, mg/mL	
		MIC	MBC/MFC
Gram-positive bacteria			
*Staphylococcus aureus*	11.0 ± 0.5 ^b^	37.5 ± 1.5 ^b^	75.0 ± 2.0 ^b^
*Bacillus cereus*	12.0 ± 0.0 ^b^	18.7 ± 1.0 ^a^	37.5 ± 1.5 ^a^
Gram-negative bacteria			
*Escherichia coli*	12.0 ± 0.0 ^b^	37.5 ± 1.3 ^b^	75.0 ± 2.5 ^b^
*Salmonella Abony*	10.0 ± 0.5 ^a^	75.0 ± 1.5 ^c^	150.0 ± 5.0 ^c^
*Pseudomonas aeruginosa*	14.0 ± 0.5 ^c^	37.5 ± 1.5 ^b^	75.0 ± 2.5 ^b^
Yeast			
*Candida albicans*	19.0 ± 0.5 ^d^	18.7 ± 0.6 ^a^	37.5 ± 2.0 ^a^

Different letters (^a–d^) designate statistically different results (*p* ≤ 0.05). MIC = minimum inhibitory concentration; MBC = minimum bactericidal concentration; MFC = minimum fungicidal concentration.

**Table 6:** Sensory, physicochemical, texture parameters and color indicators of pumpkin powder shortbread cookies (results are presented as mean ± standard deviation).

Indicators/ Parameters	Shortbread Cookies				
	CS	5% YPP	10% YPP	15% YPP	20% YPP
Sensory indicators					
Average score of sensory profile	4.59 ± 0.04 ^a,b^	4.68 ± 0.02 ^c^	4.77 ± 0.01 ^d^	4.90 ± 0.02 ^e^	4.55 ± 0.05 ^a,b^
Consistency	4.64 ± 0.02 ^b^	4.67 ± 0.03 ^b,c^	4.72 ± 0.02 ^c^	4.87 ± 0.01 ^d^	4.48 ± 0.01 ^a^
Taste	4.75 ± 0.01 ^b^	4.84 ± 0.02 ^c^	4.94 ± 0.02 ^d,e^	4.99 ± 0.01 ^e^	4.62 ± 0.02 ^a^
Appearance	4.66 ± 0.01 ^a^	4.69 ± 0.02 ^a^	4.82 ± 0.0 ^b^	4.96 ± 0.02 ^c^	4.66 ± 0.01 ^a^
Color	4.46 ± 0.03 ^a,b^	4.63 ± 0.02 ^c^	4.75 ± 0.0 ^d^	4.83 ± 0.01 ^e^	4.51 ± 0.02 ^b^
Odor	4.45 ± 0.02 ^a^	4.56 ± 0.02 ^b^	4.61 ± 0.0 ^b^	4.84 ± 0.02 ^c^	4.45 ± 0.20 ^a^
Physicochemical indicators					
Moisture content, %	2.93 ± 0.053 ^a^	3.37 ± 0.57 ^b^	3.5 ± 0.21 ^c^	3.67 ± 0.088 ^c,d^	3.87 ± 0.082 ^e^
Swelling in water, %	183.6 ± 1.4 ^c^	166.8 ± 0.0 ^b^	165.5 ± 1.4 ^b^	163.6 ± 1.0 ^a,b^	161.8 ± 0.5 ^a^
Water activity (a_w_), c. u.	0.177 ± 0.001 ^a^	0.177 ± 0.001 ^a^	0.176 ± 0.001 ^a^	0.183 ± 0.001 ^b^	0.199 ± 0.001 ^c^
Texture parameters of cookie doughs					
Hardness, g	1923.4 ± 45.7 ^c^	893.2 ± 17.8 ^b^	670.6 ± 12.4 ^a^	630.0 ± 25.3 ^a^	618.5 ± 31.5 ^a^
Adhesiveness, g·s	1639.0 ± 26.2 ^c^	742.2 ± 18.9 ^b^	560.5 ± 21.3 ^b^	514.1 ± 32.6 ^a,b^	503.7 ± 17.4 ^a^
Cohesiveness, %	0.200 ± 0.001 ^a^	0.221 ± 0.001 ^b^	0.221 ± 0.001 ^b^	0.221 ± 0.001 ^b^	0.221 ± 0.001 ^b^
Resilience, %	0.102 ± 0.001 ^d^	0.094 ± 0.001 ^c^	0.093 ± 0.001 ^b,c^	0.089 ± 0.0 ^a^	0.088 ± 0.001 ^a^
Gumminess, g	386.7 ± 8.2 ^c^	196.5 ± 9.1 ^b^	147.5 ± 14.1 ^a^	138.6 ± 15.7 ^a^	136.1 ± 11.3 ^a^
Texture parameters of cookies					
Hardness, g	1265.8 ± 31.4 ^a^	1991.7 ± 26.5 ^b^	2321.7 ± 19.4 ^c^	2435.3 ± 22.7 ^c^	2539.9 ± 28.4 ^d^
CIELab chromatic parameters of shortbread cookie cores					
L*	75.10 ± 0.45 ^d^	67.17 ± 0.48 ^c^	61.89 ± 0.03 ^b^	56.56 ± 0.65 ^a^	57.28 ± 0.28 ^a^
a*	0.66 ± 0.15 ^a^	0.11 ± 0.06 ^a^	3.07 ± 0.12 ^b^	5.60 ± 0.08 ^c^	6.19 ± 0.12 ^c^
b*	27.01 ± 0.18 ^a^	31.21 ± 0.07 ^b^	34.54 ± 0.08 ^b,c^	35.39 ± 0.08 ^d^	35.99 ± 0.07 ^d^
ΔE*	-	8.99 ± 0.41 ^a^	15.39 ± 0.58 ^b^	20.94 ± 0.32 ^c,d^	20.71 ± 0.67 ^c,d^
CIELab chromatic parameters of shortbread cookie crusts					
L*	75.39 ± 0.27 ^d^	67.24 ± 0.46 ^c^	60.91 ± 0.06 ^b^	56.08 ± 0.84 ^a^	55.23 ± 0.19 ^a^
a*	1.42 ± 0.07 ^a,b^	2.33 ± 0.04 ^b^	4.16 ± 0.08 ^c^	6.01 ± 0.05 ^d^	8.71 ± 0.05 ^e^
b*	31.82 ± 0.23 ^a^	34.54 ± 0.11 ^b^	36.70 ± 0.12 ^c,d^	37.73 ± 0.19 ^d,e^	38.52 ± 0.14 ^d,e^
ΔE*	-	8.64 ± 0.43 ^a^	15.52 ± 0.31 ^b^	20.71 ± 0.57 ^c^	22.46 ± 0.64 ^c,d^

Different letters (^a–e^) designate statistically different results (*p* ≤ 0.05). L*—luminosity; a*—red/green component; b*—yellow/blue component; ΔE*—overall difference of color; CS—control sample; YPP—shortbread cookies with yellow pumpkin powder.

## Data Availability

No new data were created or analyzed in this study. Data sharing is not applicable to this article.
